# Evaluation of the inter and intraobserver reproducibility of the “defect coverage index method”, a new computed tomography assessment method of sagittal graft positioning in arthroscopic bone block procedures

**DOI:** 10.1186/s40634-023-00590-3

**Published:** 2023-03-30

**Authors:** Cristina Delgado, Ignacio De Rus, Pablo Cañete, Jorge Díaz, Raquel Ruiz, Miguel García Navlet, Miguel Ángel Ruiz Ibán

**Affiliations:** 1 Hospital Universitario Fundación Jiménez Díaz, Madrid, Spain; 2 Hospital Universitario Torrejón, Madrid, Spain; 3 Hospital Manises, Valencia, Spain; 4 Shoulder and Elbow Reconstructive Surgery Unit. Department of Orthopaedic Surgery and Traumatology, Hospital Universitario Ramón y Cajal, Cta Colmenar km 9,100, 28046 Madrid, Spain; 5 Hospital Asepeyo Coslada, Madrid, Spain

**Keywords:** Shoulder instability, Glenoid bone defect, Bone block procedure, Reproducibility, Sagittal position, Computed tomography

## Abstract

**Purpose:**

To assess the reproducibility of a new 2-dimensional computed tomography (CT) method of assessing graft positioning in arthroscopic bone block procedure.

**Methods:**

This is a prospective observational study. Twenty-seven patients, (all men, mean [Standard deviation] age at surgery 30.9 [8.49] years) were included. Vertical graft position was assessed on the sagittal view by measuring the amount of glenoid bone defect covered by the graft. The length of the bone defect and the amount of graft covering the defect were measured. Positioning of the graft on the sagittal plane was classified as accurate if the graft covered at least 90% of the defect. Intraobserver and interobserver reproducibility was analyzed using intraclass correlation coefficients (ICC) and Kappa coefficient with 95% confidence.

**Results:**

Excellent intraobserver reproducibility was found, with an ICC of 0.94 (CI 95%, 0.86-0.97). Interobserver reproducibility was good, with the ICC value of 0.71, ranging from 0.45 to 0.86 (CI 95%).

**Conclusion:**

This new method of assessing graft positioning in arthroscopic bone block procedures on 2-dimensional computed tomography scans is reliable, with an excellent intraobserver and good interobserver reproducibility.

**Level of evidence:**

III

## Introduction

Glenoid bone loss has been reported in up to 90% of cases of recurrent anterior shoulder instability [1]. Glenoid bone deficiency shortens the glenoid arc length, reducing the glenoid surface and its concavity [2]. Hence, glenoid bone loss has been identified as a risk factor for recurrent shoulder dislocations or failure of a soft tissue repair [3]. The management of anterior shoulder instability with glenoid bone loss may require a bone grafting procedure [4]. Different bone grafting procedures have been described including coracoid transfer techniques as well as free bone grafting techniques. The bone block technique is an arthroscopic procedure that provides an anatomical reconstruction, performed through the classical portals used for the arthroscopic Bankart repair [5]. This technique allows for accurate positioning of the graft, preserves the integrity of the subscapularis tendon, and avoids damage to neurovascular structures, at risk during Latarjet procedures [6].

The success of the glenoid area reconstruction procedures largely depends on the accurate positioning of the graft [7] in both the axial and sagittal plane. Too medial position of the graft may lead to recurrence, whereas an excessively lateral position may result in the development of osteoarthritis. Furthermore, too high positioning in the sagittal plane is associated with an increased incidence of failure and recurrence of instability [8]. It is thus necessary to have a reliable method for assessing the graft position. However, there are no reproducible methods to assess sagittal graft position for arthroscopic bone block procedures, as the methods developed to assess the coracoid position after a Latarjet procedure do not take into account the defect position [9].

The purpose of this study is to describe a new 2-dimensional computed tomography (CT) method of assessing graft position in arthroscopic bone block procedure and to assess the intra and interobserver reproducibility of the method.

## Methods

The study was approved by the local Institutional Review Board (Approval number 366-20) and oral and written consent for participation was obtained for each subject.

We conducted a prospective, multicentric study of patients with anterior glenohumeral instability and glenoid bone loss who were managed surgically with an arthroscopic bone block procedure using iliac crest autograft or allograft from 2016 to 2020.

The inclusion criteria for the participants were: (1) 18 years or older, (2) recurrent anterior shoulder instability, (3) anterior glenoid bone loss affecting at least 5% of the glenoid surface area, (4) that had undergone an arthroscopic bone block procedure, and (5) that, after being informed about the study design, were able to understand and consented to participate.

All subjects had a CT scan performed preoperatively and a second CT scan performed one to 3 months postoperatively to assess the position of the graft. Measurements were performed by two investigators to determine the interobserver reproducibility. Both observers repeated their measurements 1 month later to determine intraobserver reproducibility.

All surgical procedures were performed by two orthopedic surgeons with more than 10 years of experience in shoulder surgery using the arthroscopic technique described by Taverna et al. [5, 10]. Briefly, a tricortical iliac crest bone graft is shaped to measure 20x10x10 cm. The labrum is detached from the glenoid rim and the glenoid defect is decorticated and flattened. The bone block is introduced and positioned flush against the glenoid rim. Fixation is obtained with two round Endobuttons (Smith & Nephew Inc., Andover, MA, USA). Tension of 100 N is applied for both implants. Finally, the capsule-labrum complex is re-attached to the glenoid with anchors, leaving the graft extra-articular.

### The “Defect Coverage Index Method” for the assessment of sagittal graft position

The CT scans of all patients were anonymized and downloaded to a computer. The radiological evaluation was performed using Horos (Pixmeo 3.3.6 version, Switzerland).

**Fig. 1:**
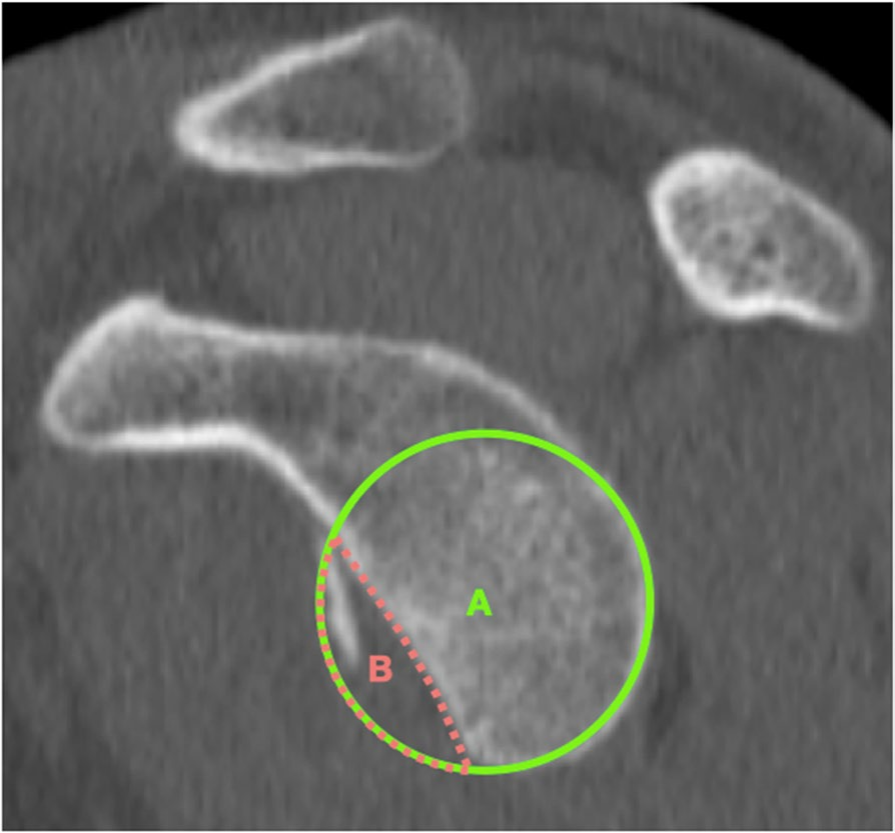
a CT en-face image of the left shoulder of a subject with anterior shoulder instability and apparent anterior glenoid bone loss. The glenoid bone defect is measured using the PICO method. A best-fit circle is drawn around the posteroinferior cortical margin of the glenoid. The defect is manually drawn and measured using the area tool

First, in the preoperative CT scan, an en-face view of the glenoid was obtained. With 3D multiplanar reconstruction (MPR) mode a simultaneous vision of axial, sagittal and coronal planes was obtained. Maintaining working axes parallel to the glenoid surface area an en-face view of the glenoid was obtained [11]. The glenoid bone defect is assessed in the en-face view using the PICO method [12] (Fig. 1).

**Fig. 2:**
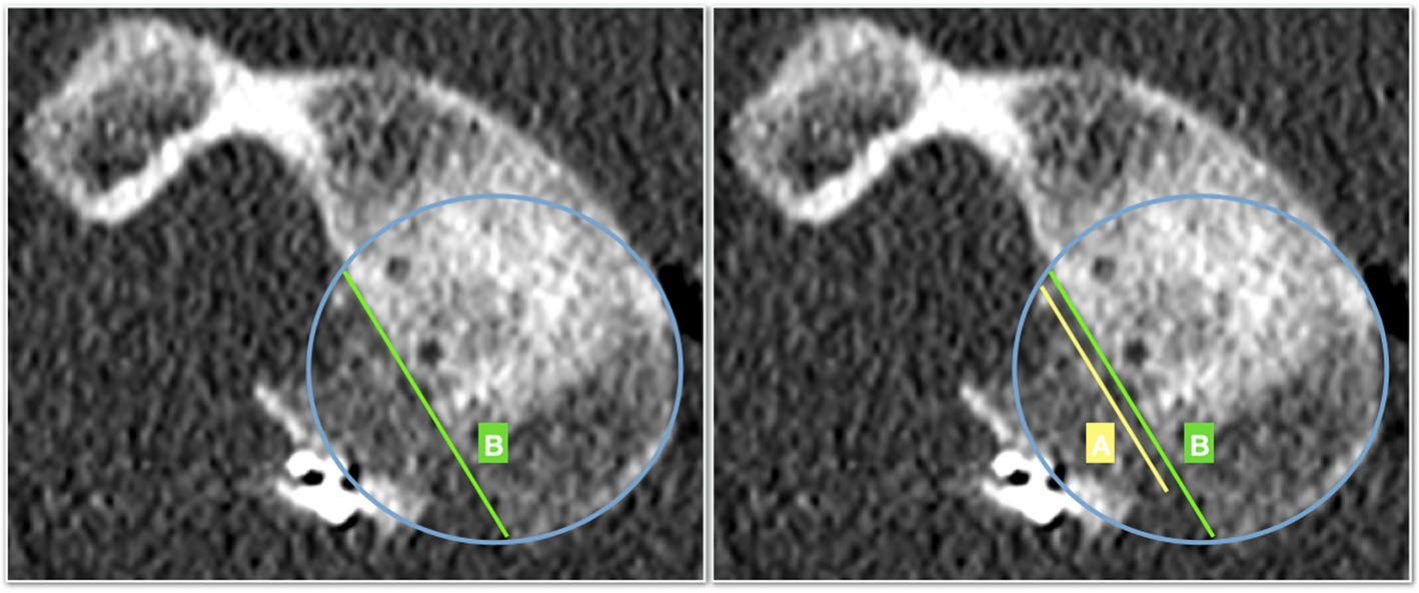
a postoperative CT en-face image of the left shoulder of a subject with anterior shoulder instability following an arthroscopic bone block procedure. The bone-block position is evaluated using the “Defect coverage index method”. First the length of the bone defect is measured (green line, B) (A). Secondly, the amount of graft covering the glenoid defect is quantified

**Fig. 3:**
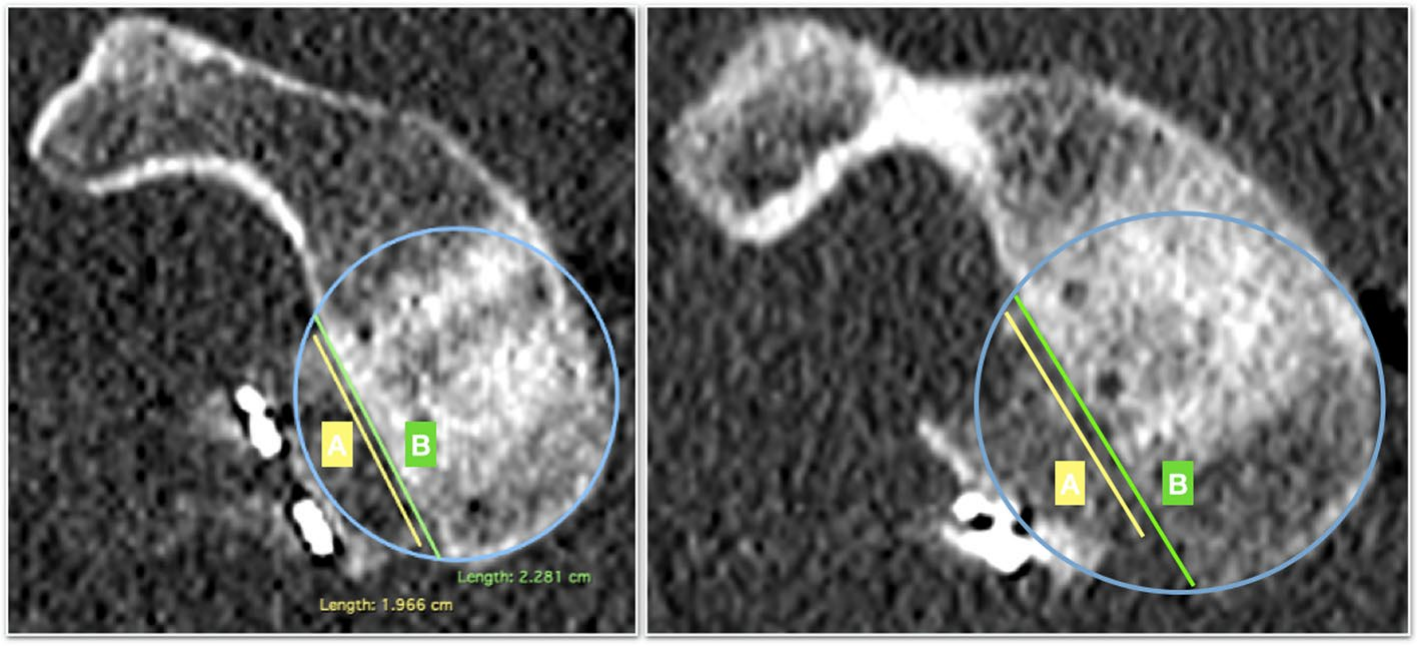
**A** In this example, the bone block covers more than 90% of the defect and can be considered accurate positioned. **B** In this example, less than 90% of the defect is covered by the graft. The bone-block can’t be considered as well-positioned

Second, in the postoperative CT scan the sagittal plane graft’s position is assessed on the en-face view by measuring the amount of glenoid bone defect covered by the graft (Fig. 2). The extension of the bone defect is confirmed with the preoperative CT data. The length of the bone defect (B) and the amount of graft covering the defect (A) are measured and the percentage of coverage of the bone defect is calculated. Positioning of the graft on the sagittal plane was arbitrarily classified as precise if the graft covered at least 90% of the defect (Fig. 3).

### Statistical analysis

The normality of the quantitative variables included in the study was analyzed with the Kolmogorov-Smirnoff goodness-of-fit test. Patient’s characteristics were analyzed using means and standard deviation for quantitative variables and using frequencies and percentages for qualitative variables. The reproducibility of the measurement was assessed using two Model intraclass correlation coefficients (ICC) [13]. ICC below 0.5 were considered poor, between 0.5 and 0.75 moderate, 0,75 to 0.9 good and > 0.9 excellent [14]. For intraobserver reproducibility the data from both measurement sessions of each investigator were compared. For interobserver reproducibility the data from the two investigators were compared. Bland-Altmann plots were obtained for all six variables [15]. The significance level was established at *p* < 0.05. No beforehand sample size calculation was performed.

An arbitrary cut-off point of at least 90% coverage of the defect was established to further assess reproducibility: the percentage of correctly positioned grafts is calculated.

## Results

A total of 27 consecutive patients, all male with a mean ± standard deviation age at surgery of 30.9 ± 8.49 years were evaluated and included in the study. The dominant arm was involved in 17 (63%). Twenty patients (74.1%) participated in sports: 13 were enrolled in high-risk sports involving collision or overhead activities, three at the competitive level. Pre-operative glenoid bone loss was 13.7 ± 3.33%. A Hill-Sachs lesion in all subjects.

An iliac crest allograft was used in 13 (52%) patients, and iliac crest autograft in 12 (48%). Capsulolabral repair was done in all cases. An associated remplissage procedure was performed in 20 (74.1%) patients. No intraoperative or immediate postoperative complications occurred.

The postoperative CT scan was performed 2.4 ± 0.7 months postoperatively.

The two examiners analyzed all CT scans independently twice with a 1-month interval. The mean coverage of the defect was 98.6 ± 16.9% (range 73.7-100%) for the first time and 94.5 ± 16.9% (range 67-100) for the second time (Table 1). These measurements showed excellent intraobserver reproducibility, with an ICC of 0.94 (CI 95%, 0.86-0.97). Graft placement was considered accurate in 74.1% (20/27) patients at first measurement and 70.4% (19/27) at second evaluation. The Kappa coefficient for intraobserver reproducibility was moderate (K = 0.72).

**Table 1:** Coverage ratio following first and second measurement in both assessors

	Observer 1	Observer 2	ICC
Measurement 1	98.6 ± 16.9%	94 ± 2639%	Interobserver 0.71
Measurement 2	94.5 ± 16.9%	96.4 ± 8.9%	
Mean	97.8% ± 7.67	95.2 ± 8.29%	
ICC	Intraobserver 0.94		

Interobserver reproducibility about sagittal bone block location was moderate, with the ICC value of 0.71 (CI 95%, 0.45-0.86). The first observer noted a mean coverage of 97.8% ± 7.67 (range 70.3-100). The mean coverage of the defect was 95.2 ± 8.29% (range 67.7-100) according to the second assessor.

When assessing whether the graft was correctly positioned using the 90% coverage threshold, the two examiners had moderate interobserver agreement (Kappa 0.59) (Table 2).

**Table 2:** Percentage of subjects with adequately positioned grafts

	Observer 1	Observer 2	Kappa
Measurement 1	74.1% (20/27)	77.8% (21/27)	Interobserver 0.59
Measurement 2	70.4% (19/27)	85.2% (23/27)	
Kappa	Intraobserver 0.72		

## Discussion

The main finding of this study is that the method presented here for the assessment of the sagittal graft position in arthroscopic bone block procedure is reliable, showing good intraobserver and interobserver reproducibility.

The success of shoulder stabilization procedures with the bone block technique is largely dependent on the correct positioning of the bone graft [16]. For the Latarjet procedure the sagittal position is commonly assessed measuring the amount of graft located above and below the glenoid equator [9]. Kraus et al. have shown that this method is reliable, with good interobserver and intraobserver reproducibility [7]. It is believed that the graft should be placed at the glenoid equator or below it [17]. Grafts positioned above the equator are more prone to recur, whereas very low located grafts may be susceptible to mechanical failure [18, 19]. Positioning the graft below the equator is crucial for the triple locking effect of the Latarjet procedure. Patte et al. [20] introduced the concept of triple locking, which includes the restoration of the glenoid surface with the coracoid graft; the sling effect, a dynamic restraint provided between the conjoined tendon and the inferior part of the subscapularis tendon; and the capsular effect after suturing of the capsule with the coracoacromial ligament [21]. According to Patte [20] the graft should be placed anteroinferior where the glenoid defect is usually located. Furthermore, the sling effect works most effectively when placing the graft below the equator [22].

However, the stabilizing mechanism of bone block procedures does not depend on this triple effect. The main purpose of these procedures is to cover de bone defect, thus, restoring the glenoid surface area and glenoid concavity [19, 23]. Therefore, when assessing graft’s position in bone block procedures it is important to evaluate the coverage of the defect and not whether the graft is above or below the equator. Hence, the evaluation of the graft considering its position related to the equator may be inadequate to properly assess arthroscopic bone block procedures.

There are few studies investigating the positioning of the postoperative graft in arthroscopic bone block procedures. Different authors use the same technique as for the evaluation of the coracoid graft in Latarjet procedure (that is, considering its position related to the equator): Taverna et al. [6] reported that 92.3% of bone blocks (26 of 26) were optimally positioned in the sagittal plane. Boileau et al. [24] evaluated 7 patients after an arthroscopic bone block technique in the setting of revision of patients with failed Latarjet repair. In 100% of patients the graft was optimally positioned in the sagittal plane.

Results of sagittal positioning in arthroscopic bone block procedure evaluated with the new method described above have recently been reported by Delgado et al. [25]. In their series of 25 patients, 80% of the grafts (20 of 25) were well positioned in the sagittal plane.

This study has some limitations. First, the sample is relatively small, and no formal determination of the sample size was made. However arthroscopic iliac crest bone grafting is still a not very common procedure [26]. Moreover, when analyzing bone block procedure results few studies are available, and its sample is also small. Second, measurements were manually drawn. However, based on the observed ICC, all measurements were highly reliable. Furthermore, the fact that all measurements were performed by the same assessor may limit the variability of the measurements. Finally, clinical validation of the method will be needed to assess if the presented cut-off point of at least 90% coverage is correlated with clinical outcomes.

## Conclusion

The new proposed method to assess the sagittal graft position after arthroscopic bone block procedures on 2-dimensional computed tomography scans is reliable, with an excellent intraobserver and moderate interobserver reproducibility.
